# Delayed Diagnosis of Aortic Dissection: A Scoping Review

**DOI:** 10.31083/RCM33487

**Published:** 2025-06-25

**Authors:** Yaru Xiao, Sufang Huang, Danli Zheng, Ying Li, Jian Ke, Xiaorong Lang, Danni Feng

**Affiliations:** ^1^Emergency Department, Tongji Hospital, Tongji Medical College, Huazhong University of Science and Technology, 430030 Wuhan, Hubei, China; ^2^Nursing Department, Tongji Hospital, Tongji Medical College, Huazhong University of Science and Technology, 430030 Wuhan, Hubei, China

**Keywords:** aortic dissection, delayed diagnosis, scoping review

## Abstract

**Background:**

Patients with aortic dissection (AD) exhibit an elevated early mortality rate. A timely diagnosis is essential for successful management, but this is challenging. There are limited data delineating the factors contributing to a delayed diagnosis of AD. We conducted a scoping review to assess the time to diagnosis and explore the risk factors associated with a delayed diagnosis.

**Methods:**

This scoping review was conducted in accordance with the Preferred Reporting Items for Systematic Reviews and Meta-Analyses (PRISMA) guidelines. We conducted online searches in PubMed, Web of Science, Cochrane Library, Bing, Wanfang Data Chinese database, and the China National Knowledge Infrastructure (CNKI) Chinese database for studies that evaluated the diagnostic time and instances of delayed diagnoses of AD.

**Results:**

A total of 27 studies were retrieved from our online searches and included in this scoping review. The time from symptom onset to diagnosis ranged from 40.5 min to 84.4 h, and the time from hospital presentation to diagnosis ranged from 0.5 h to 25 h. Multiple factors resulted in a significantly delayed diagnosis. Demographic and medical history predictors of delayed diagnosis included the female sex, age, North American versus European geographic location, initial AD, history of congestive heart failure, history of hyperlipidemia, distressed communities index >60, walk-in visits to the emergency department, those who transferred from a non-tertiary care hospital, and preoperative coronary angiography. Furthermore, chest and back pain, especially abrupt or radiating pain, low systolic blood pressure, pulse deficit, and malperfusion syndrome required less time for diagnostic confirmation. In contrast, painlessness, syncope, fever, pleural effusion, dyspnea, troponin positivity, and acute coronary syndrome-like electrocardiogram were more prevalent in patients with a delayed diagnosis.

**Conclusions:**

A recognition of the features associated with both typical and atypical presentations of AD is useful for a rapid diagnosis. Educational efforts to improve clinician awareness of the various presentations of AD and, ultimately, improve AD recognition may be relevant, particularly in non-tertiary hospitals with low exposure to aortic emergencies.

## 1. Introduction

An aortic dissection (AD) is a critical tear in the aortic intimal layer that leads to dissection of the aortic wall. The Stanford criteria categorizes ADs into type A which involves the ascending aorta, and type B which does not [1]. Acute aortic dissection (AAD) is defined as a dissection occurring within 14 days of the onset of pain [2]. AAD is a critical disease that requires quick and accurate diagnosis because a delay in treatment carries a high mortality rate [3, 4]. The mortality rate for AAD within the first 24 to 48 hours following the onset of symptoms is described as 1% to 2% per hour based on data from the 1950s [5, 6]. The International Registry of Acute Aortic Dissection (IRAD) updated the data in 2022, and non-operative patients presenting with type A acute aortic dissection (TAAAD) had a mortality in the first 48 hours of 0.5% per hour [6]. However, not all patients with AAD present with the typical onset of severe chest or back pain. Some patients exhibit neurological deficits, dyspnea, or other symptoms [3, 5, 7]. AAD has symptoms similar to those of other diseases, making diagnosis difficult. Therefore, AAD is highly susceptible to misdiagnoses, such as acute coronary syndrome (ACS), as well as neurological, pulmonary, and gastrointestinal diseases [8, 9]. Studies have shown that 15%–39% of patients are misdiagnosed at initial diagnosis [8, 9, 10, 11, 12]. In a study by Spittell *et al*. [7] on 236 patients, 38% were misdiagnosed at the time of the initial visit, and 28% of these misdiagnosed patients were only definitively diagnosed at the time of autopsy. In addition, it is reported that 16.5%–17.6% of AAD patients were missed during emergency department (ED) visits [13, 14]. Missed diagnoses and misdiagnoses usually delay the diagnosis. An early diagnosis and initiation of intervention in AAD limits the risks of aortic rupture, cardiac tamponade, and death.

Therefore, it is essential to review the literature to investigate the risk factors of delayed diagnosis. The purpose of this scoping review was to determine and summarize what is known about the delayed diagnosis of AAD, specifically regarding diagnosis time and the risk factors of delayed diagnosis.

## 2. Methods

A scoping methodology was used to explore the breadth of the literature available about the delayed diagnosis of AD. Scoping reviews lead to recommendations for future research, aiming to provide contextual knowledge and identify existing literature gaps [15, 16]. Scoping reviews allow for analytic frameworks or thematic development. The Arksey and O’Malley framework uses five stages to conduct a scoping review: (1) identifying the research question; (2) identifying the relevant studies; (3) study selection; (4) charting the data; and (5) collating, summarizing, and reporting the results [17].

### 2.1 Identifying the Research Question

The first stage of the Arksey and O’Malley framework requires identification of an area of interest and an exploration of these concepts [17]. This stage of the scoping review framework aims to guide the search strategy. The research questions pertinent to this review were as follows:

What is the diagnostic time in patients with AD?

What factors influence diagnostic delay in patients with AD?

### 2.2 Identifying the Relevant Studies

To ensure that sufficient information was obtained, the following databases were searched: PubMed, Web of Science, Cochrane Library, Bing, Wanfang Chinese, and Zhiwang or China National Knowledge Infrastructure Chinese database. The search strategy included a combination of the National Library of Medicine Medical Subject Headings (MeSH), in addition to exploring key words representing the concepts of “aortic dissection”, “diagnostic time”, and “diagnostic delay”. There were no restrictions on the language, date, or type of study.

### 2.3 Study Selection

#### 2.3.1 Data Management, Screening, and Extraction

**Fig. 1.**
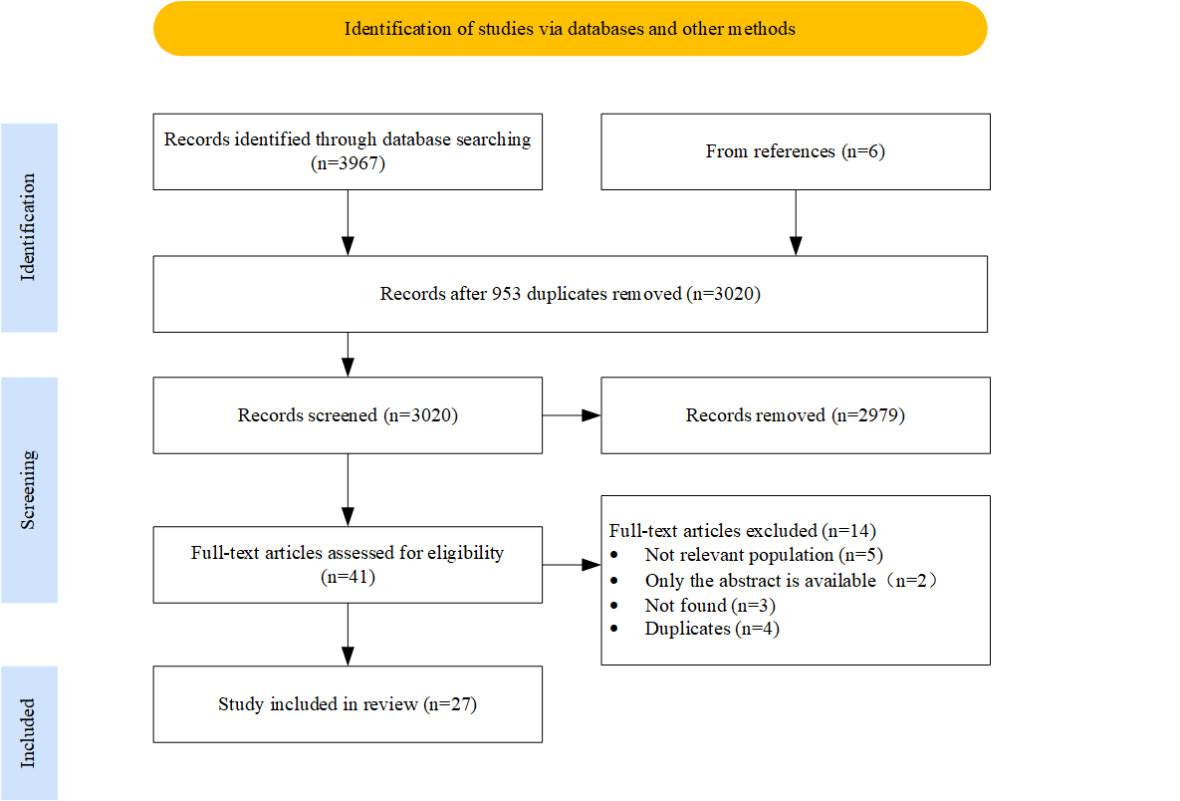
**Preferred Reporting Items for Systematic Reviews and Meta-Analyses (PRISMA) flow diagram**.

The retrieved articles were imported into the Endnote citation management system, and duplicates were eliminated. Microsoft Excel software was used to screen the titles, abstracts, and full text of retrieved articles. Initially, titles and abstracts were screened by two independent authors to exclude irrelevant studies. Subsequently, the two authors independently read the full text of retrieved articles to determine inclusion. A third reviewer adjudicated in case of disputes over the inclusion of a study. Two authors extracted the data from the included articles. Finally, 27 studies were included in this scoping review. The process of identification, screening, eligibility, and inclusion of the studies is shown in Fig. 1.

The process of searching on PubMed:

#1((((((((((((((Aortic Dissection[MeSH Terms]) OR (Aortic Dissections[Title/Abstract])) OR (Dissection, Aortic[Title/Abstract])) OR (Aortic Dissecting Aneurysm[Title/Abstract])) OR (Aneurysm, Aortic Dissecting[Title/Abstract])) OR (Aortic Dissecting Aneurysms[Title/Abstract])) OR (Dissecting Aneurysm, Aortic[Title/Abstract])) OR (Dissecting Aneurysm Aorta[Title/Abstract])) OR (Aortic Syndrome[Title/Abstract])) OR (Aneurysm Aorta, Dissecting[Title/Abstract])) OR (Aorta, Dissecting Aneurysm[Title/Abstract])) OR (Dissecting Aneurysm Aortas[Title/Abstract])) OR (Aneurysm, Dissecting[Title/Abstract])) OR (Dissecting Aneurysms[Title/Abstract])) OR (Dissecting Aneurysm[Title/Abstract]).

#2(((((((((((((Delayed Diagnosis[MeSH Terms]) OR (Delayed Diagnoses[Title/Abstract])) OR (Diagnosis, Delayed[Title/Abstract])) OR (Diagnosis Delay[Title/Abstract])) OR (Diagnosis Delays[Title/Abstract])) OR (Late Diagnosis[Title/Abstract])) OR (Diagnosis, Late[Title/Abstract])) OR (Late Diagnoses[Title/Abstract])) OR (Delay*[Title/Abstract])) OR (diagnostic time*[Title/Abstract])) OR (Time to diagnosis[Text Word])) OR (symptom onset to diagnosis[Text Word])) OR (presentation to diagnosis[Text Word])) OR (admission to diagnosis[Text Word]).

#3 #1 AND #2

#### 2.3.2 Inclusion and Exclusion Criteria

Articles meeting the following conditions were included in this review: research articles that included diagnostic time or delayed diagnosis in patients with AD; there were no restrictions on publication date or type of research article. Articles meeting the following conditions were excluded from this review: (1) full text of the selected article was unavailable; and (2) repetitive studies. All relevant studies published up to December 2023 were retrieved.

### 2.4 Charting the Data

#### 2.4.1 Critical Appraisal

According to the guidelines for systematic scoping of reviews [18], the objective was to determine the scope and type of literature; therefore, no quality assessment was conducted.

#### 2.4.2 Data Collection and Synthesis

The following data were extracted and classified: author, publication year, study design, patient period, patient source, type of patients, number of patients, diagnostic time, risk factors, and original explanations.

### 2.5 Collating, Summarizing, and Reporting the Results

A total of 3967 records were found by searching the databases; 953 duplicate records were deleted, and 27 records met the inclusion criteria according to the screening process. The screening process and the reasons for excluding studies are presented in Fig. 1.

## 3. Results

A total of 27 studies were included in the analysis after reviewing all potentially relevant studies identified via our online searches.

### 3.1 Diagnostic Time

There was a total of 27 articles included in this review of the diagnostic time for patients with AD (Table 1, Ref. [2, 5, 6, 9, 10, 11, 19, 20, 21, 22, 23, 24, 25, 26, 27, 28, 29, 30, 31, 32, 33, 34, 35, 36, 37, 38, 39]). Table 1 shows the details regarding first author, publication year, study design, patient period, patient source, patient type, number of patients, and diagnostic time. The time from symptom onset to diagnosis ranged from 40.5 min to 84.4 h, and the time from hospital presentation to diagnosis ranged from 0.5 h to 25 h.

**Table 1.** **Diagnostic times of AD**.

First author, publication year	Study design	Patients period	Source of patients	Type of patients	No. of patients	Time from symptom onset to diagnosis	Time from hospital presentation to diagnosis
Park 2004 [5]	Retrospective cohort study	1997 to 2001	IRAD	AAD	977	Patients having no pain, 29 h;	-
						Having pain, 10 h	
Upchurch 2005 [19]	Retrospective cohort study	1996 to 2001	IRAD	AAD	992	Patients presenting primarily with abdominal pain, 84.4 ± 193.2 h;	-
						All others, 50.4 ± 97.8 h	
Hansen 2007 [10]	Retrospective study	2000 to 2004	St. Michael’s hospital, Canada	AAS	66	29 ± 7 h	-
Rapezzi 2008 [20]	Retrospective cohort study	1996 to 2006	Metropolitan Hospital, Italy	AAD	161	330 min, IQR 893	177 min, IQR 644
Raghupathy 2008 [21]	Retrospective cohort study	January 1, 1996, to November 20, 2004	IRAD	TAAAD	615	North America, 15.3 (4.4–48.0) h; Europe, 6.0 (3.0–24.0) h	-
Harris 2010 [22]	Prospective and retrospective cohort study	January 1, 2003 to July 31, 2005	Abbott Northwestern Hospital, USA	AAD	30	-	279 (109, 945) min
Harris 2010 [22]	Prospective and retrospective cohort study	August 1, 2005 to September 1, 2009	Abbott Northwestern Hospital, USA	AAD	71	-	160 (82, 288) min
Kurabayashi 2011 [11]	Retrospective cohort study	April 2005 to March 2010	The Yokohama City Minato Red Cross Hospital, Japan	AAD	109	-	Diagnosed patients, 1 (1.0) h;
							Misdiagnosed patients, 25 (59.0) h
Ramanath 2011 [23]	Retrospective cohort study	January 27, 1996, to May 3, 2010	IRAD	TAAAD	1343	Preoperative coronary angiography patients,14.3 (4.5–68.3) h;	-
						Nonpreoperative coronary angiography patients, 8.5 (3.4–26.5) h	
Harris 2011 [2]	Retrospective study	January 1, 1996, to January 29, 2007	IRAD	TAAAD	894	-	4.3 (1.5–24) h
Imamura 2011 [24]	Retrospective cohort study	2002 to 2007	Shinshu University School of Medicine, Matsumoto, Japan	AAD	98	-	Painless group, 2.0 h;
							Painful group, 0.5 h
Tolenaar 2013 [25]	Retrospective cohort study	January 1996 to July 2012	IRAD	TBAAD	1162	Painless patients, 34.0 (22.8–72) h;	-
						Not painless patients, 19.0 (12.7–25.3) h	
Bossone 2013 [26]	Retrospective cohort study	January 3, 1996 and February 12, 2011	IRAD	AAD	1354	-	Overall, 3.0 (1.0–13.0) h;
							White, 3.0 (0.9–12.9) h;
							Black, 3.5 (1.3–13.8) h
Du 2015 [27]	Retrospective cohort study	January 2005 to July 2014	Beijing Tongren Hospital, China	AAD	96	12 h 40 min	-
Pare 2016 [28]	Retrospective cohort study	March 2013, to May 2015	3 affiliated Hospitals, USA	AAD	32	-	EP FOCUS, 80 (46–157) min;
							non-EP FOCUS, 226 (109–1449) min
Hirata 2015 [9]	Retrospective cohort study	1983 to 2011	Okinawa Chubu Hospital, Japan	TAAAD	127	-	1.5 (0.5–4.0) h
Vagnarelli 2015 [29]	Retrospective cohort study	2000 to 2013	AESA, Italy	AAS	398	Overall, 307 (180–900) min;	Overall, 166 (90–353) min;
						AHF, 333 (180–1112) min;	AHF, 209 (92–510) min;
						No AHF, 300 (193–840) min	No AHF, 160 (86–322) min
Vagnarelli 2016 [30]	Retrospective cohort study	2000 to 2013	AESA, Italy	AAS	248	Overall, 347 (195–895) min;	Overall, 190 (101–406) min;
						Abnormal troponin T values, 439 (197–1500) min;	Abnormal troponin T values, 210 (103–829) min;
						Normal troponin T values, 313 (195–725) min	Normal troponin T values, 177 (100–342) min
Isselbacher 2016 [31]	Retrospective cohort study	December 26, 1995, and February 6, 2013	IRAD	AAD	3828	Overall, 5.2 (3.0–13.5) h;	-
						Initial AD, 5.3 (3.0–13.6) h;	
						Recurrent AD, 3.8 (2.2–8.9) h	
Strauss 2017 [32]	Retrospective cohort study	March 2003 and March 2013	Abbott Northwestern Hospital, USA	AAD	162	-	Early diagnosis group, 102 (63–168) min;
							Late diagnosis group, 903 (393–1933) min
Costin 2018 [33]	Retrospective cohort study	-	IRAD	TAAAD	2765	No Ischemia group, 5.0 (3.0–11.8) h;	-
						Ischemia group, 5.0 (2.5–14.1) h	
He 2020 [34]	Retrospective cohort study	January 2015 to January 2019	Zhangye People’s Hospital Affiliated to Hexi College, China	AD	180	Death group, 69.0 (57.0–120.0) min;	-
						Survival group, 40.5 (30.25–54.75) min	
Bruna 2020 [35]	Retrospective cohort study	2012 to 2016	RENAU Heart Surgical Centers, France	TAAAD	197	-	88 (46–241) min
Zaschke 2020 [36]	Retrospective cohort study	2012 to 2016	The German Heart Center Berlin	TAAAD	350	Initial misdiagnosis group, 4.0 (2.4–10.4) h;	Initial misdiagnosis group, 2.0 (0.8–5.1) h;
						Correct initial diagnosis group, 2.1 (1.5–3.2) h	Correct initial diagnosis group, 0.6 (0.3–1.4) h
Axtell 2020 [37]	Retrospective cohort study	2011 to 2017	Nanjing Drum Tower Hospital, China	TAAAD	641	11 (7–24) h	-
Axtell 2020 [37]	Retrospective cohort study	2011 to 2017	Massachusetts General Hospital, USA	TAAAD	150	3.5 (3.4–14.4) h	-
Saha 2021 [38]	Retrospective cohort study	January 2017 and January 2020	LMU University Hospital, Germany	TAAAD	96	-	2.1 (0.6–9.5) h
Harris 2022 [6]	Retrospective cohort study	January 1996 to November 2018	IRAD	TAAAD	5611	-	Intended surgery group, 2.5 (1.2–5.3) h;
							Medical management group, 3.5 (1.4–7.3) h
Lim 2022 [39]	Retrospective cohort study	February 2006 to February 2020	Montefiore Medical Center, USA	TAAAD	124	-	3.36 (1.83–6.63) h

AD, aortic dissection; IRAD, international registry of acute aortic dissection; AAD, acute aortic dissection; AAS, acute aortic syndrome; IQR, interquartile range; TAAAD, type A acute aortic dissection; TBAAD, type B acute aortic dissection; EP FOCUS, emergency physician-focused cardiac ultrasound; AHF, acute heart failure; AESA, Archivio Elettronico Sindromi Aortiche acute; RENAU, REseau Nord Alpin des Urgences; LMU, Ludwig Maximilian University of Munich.

### 3.2 Predictors of Delayed Diagnosis

Seven studies showed the predictors of delayed AD diagnosis using univariate analysis (Table 2, Ref. [5, 21, 23, 25, 31, 32, 40]). As shown in Table 2, the risk factors in relation to demographics and medical history were the female sex, North American versus European geographic location, initial AD, and transfer [21, 31, 32, 40]. In addition, TAAAD patients with preoperative coronary angiography (CA) were more likely to have a definitive diagnosis, as the time from symptom onset to diagnosis was longer among preoperative CA than among patients with non-preoperative CA. Preoperative CA is infrequently performed on patients with TAAAD, except, occasionally, on patients at high risk for myocardial ischemia, which may worsen the surgical outcome [23]. Regarding signs and symptoms, patients with chest and back pain, especially abrupt or radiating pain, were more frequent in the group with an early diagnosis. In contrast, syncope was more prevalent in patients with a delayed diagnosis [5, 25, 32]. These differences were deemed statistically significant.

**Table 2.** **Predictors of diagnostic delay of AD (only use univariate analysis)**.

Predictors		*p* value	First author, publication year	Study design	Patients period	Source of patients	Type of patients	No. of patients	Original explanation
Demographics and history	Female sex	0.031	Nienaber 2004 [40]	Retrospective cohort study	January 1, 1996, to November 19, 2001	IRAD	AAD	1078	Diagnosis of AD is more often delayed (not diagnosed in a timely manner, within 4 hours) in women than in men.
	Geographic differences: North Americans	<0.001	Raghupathy 2008 [21]	Retrospective cohort study	January 1, 1996, to November 20, 2004	IRAD	TAAAD	615	Time elapsed from symptom onset to confirmation of diagnosis: North Americans vs Europeans, median 15.3 hours, vs median 6.0, *p* < 0.001.
	Initial AD	0.012	Isselbacher 2016 [31]	Retrospective cohort study	December 26, 1995, and February 6, 2013	IRAD	AAD	3828	Time of symptoms to diagnosis: initial AD 5.3 h vs recurrent AD 3.8 h, *p* = 0.012.
	Transfer	0.02	Strauss 2017 [32]	Retrospective cohort study	March 2003 and March 2013	Abbott Northwestern Hospital, USA	AAD	162	Presentation to diagnosis times ≥300 minutes were termed late diagnosis.
									Patients with late diagnosis were more likely to be transferred from referral hospital.
Test	Preoperative CA	0.005	Ramanath 2011 [23]	Retrospective cohort study	January 27, 1996, to May 3, 2010	IRAD	TAAAD	1343	The time from symptom onset to diagnosis among preoperative CA patients was 14.3h (4.5–68.3) versus 8.5h (3.4–26.5) among nonpreoperative CA patients (*p* = 0.005).
Symptoms and signs	Painless	0.01	Park 2004 [5]	Retrospective cohort study	1997 to 2001	IRAD	AAD	977	Median time to diagnosis: painless AAD 29 h vs painful AAD 10 h, *p* = 0.01.
		0.006	Tolenaar 2013 [25]	Retrospective cohort study	January 1996 to July 2012	IRAD	TBAAD	1162	Hours between presentation and diagnosis: not painless 19.0 h vs painless 34.0 h, *p* = 0.006.
	Chest pain	<0.001	Strauss 2017 [32]	Retrospective cohort study	March 2003 and March 2013	Abbott Northwestern Hospital, USA	AAD	162	Presentation to diagnosis times ≥300 minutes were deemed as a late diagnosis.
	Back pain	0.02							
	Radiating pain	0.001							Chest and back pain, especially when abrupt or radiating were characteristics found more frequently in the group with early diagnosis. In contrast, syncope was more prevalent in those with delayed diagnosis.
	Abrupt onset of pain	0.008							
	Syncope	0.002							

CA, coronary angiography.

Seven of the selected studies examined predictors of delayed AD diagnosis by employing multivariate analysis, as shown in Table 3 [2, 9, 20, 27, 29, 30, 39]. There was no clear definition of the duration of delayed diagnosis. Five studies used the 75th percentile as the cutoff time, and delayed diagnosis was defined as the time from presentation to diagnosis >75th percentile [9, 20, 29, 30, 39]. The cut-off values ranged from 4.5 h to 12 h. Only Du *et al*. [27] deemed a median presentation-to-diagnosis time of more than 12 h 40 minutes to be classified as a delayed diagnosis. Using multivariate analysis, Rapezzi *et al*. [20] found that age <70 years, dyspnea, pleural effusion, systolic blood pressure (SBP) ≤105 mmHg, troponin positivity, and ACS-like electrocardiogram (ECG) results were associated with an increased likelihood of delayed diagnosis. Harris *et al*. [2] reported that delays in AD diagnosis occurred in women, those with an absence of atypical symptoms that were not abrupt or did not include chest, back, or any pain, patients with febrile diseases, those with an SBP ≥105 mmHg on admission, or those who transferred from a nontertiary care hospital. In addition, Hirata *et al*. [9] revealed that walk-in visits to the ED were the only predictors of delayed diagnosis. Du *et al*. [27] suggested that patients with dyspnea, troponin positivity, and ACS-like ECG findings were more likely to have a delayed diagnosis. In two studies by Vagnarelli *et al*. [29, 30], excessive risk was related to dyspnea, pleural effusion, troponin positivity, and a combination of troponin-positive and ACS-like ECG abnormalities, whereas back pain, pulse deficit, and SBP <90 mm Hg were protective against delayed diagnosis. In a recent study by Lim *et al*. [39], increased age, chest and back pain at presentation, evidence of malperfusion syndrome, and a history of congestive heart failure were associated with a decreased risk of delayed diagnosis. In contrast, a history of hyperlipidemia and a distressed communities index >60 were associated with an increased risk of diagnostic delay.

**Table 3.** **Predictors of diagnostic delay of AD (multivariate analysis)**.

Predictors	OR	95% CI	*p* value	First author, publication year	Study design	Patients period	Source of patients	Type of patients	No. of patients	Cut off to define delayed diagnosis
Age <70 yrs	2.34	1.03–5.36	0.043	Rapezzi 2008 [20]	Retrospective cohort study	1996 to 2006	Metropolitan Hospital, Italy	AAD	161	Time from presentation to diagnosis >75th percentile, 12 h
Dyspnea	3.33	1.29–8.59	0.013							
Pleural effusion	3.96	1.80–8.69	0.001							
SBP ≤105 mmHg	0.078	0.01–0.59	0.014							
Troponin positivity	3.63	1.12–11.84	0.03							
ACS-like electrocardiogram	2.88	1.01–8.17	0.048							
Female sex	1.73	1.27–2.36	0.001	Harris 2011 [2]	Retrospective cohort study	January 1, 1996, to January 29, 2007	IRAD	TAAAD	894	Used multiple linear regression, no definition
Transfer	3.34	2.38–4.69	<0.001							
Chest pain, posterior	1.61	0.45–0.81	0.001							
Worst pain ever	0.53	0.36–0.78	0.001							
Abrupt onset of pain	0.43	0.25–0.73	0.002							
Febrile	5.11	2.07–12.62	<0.001							
Admission SBP ≥105 mm Hg	2.45	1.80–3.33	<0.001							
Walk-in visit to the emergency room	3.72	1.39–9.9	0.009	Hirata 2015 [9]	Retrospective cohort study	1983 to 2011	Okinawa Chubu Hospital, Japan	TAAAD	127	Time from presentation to diagnosis >75th percentile, 4.5 h
Dyspnea	4.61	1.40–15.20	<0.05	Du 2015 [27]	Retrospective cohort study	January 2005 to July 2014	Beijing Tongren Hospital, China	AAD	96	Time from symptom onset to diagnosis > median, 12 h 40 min
Troponin positivity	3.66	1.29–10.37	<0.05							
ACS-like electrocardiogram	2.89	1.10–7.60	<0.05							
Back pain	0.51	0.32–0.81	0.005	Vagnarelli 2015 [29]	Retrospective cohort study	2000 to 2013	AESA, Italy	AAS	398	Time from presentation to diagnosis >75th percentile, 406 min
Pleural effusion	2.17	1.31–3.6	0.003							
Pulse deficit	0.56	0.30–1.05	0.003							
Back pain	0.51	0.31–0.86	0.01	Vagnarelli 2016 [30]	Retrospective cohort study	2000 to 2013	AESA, Italy	AAS	248	Time from presentation to diagnosis >75th percentile, 353 min
Dyspnea	2.43	1.29–4.59	0.006							
Pleural effusion	2.02	1.16–3.50	0.01							
SBP <90 mmHg	0.31	0.14–0.68	0.003							
Troponin positivity	1.92	1.05–3.52	0.03							
Positive troponin+ACS-like electrocardiogram	2.48	1.12–5.54	0.02							
Distressed communities index >60	5.108	1.519–17.174	0.008	Lim 2022 [39]	Retrospective cohort study	February 2006 to February 2020	Montefiore Medical Center, USA	TAAAD	124	Time from presentation to diagnosis >75th percentile, 6.6 h
Age	0.944	0.904–0.987	0.011							
Chest pain	0.099	0.021–0.470	0.004							
Back pain	0.247	0.083–0.734	0.012							
Malperfusion syndrome	0.040	0.007–0.241	<0.001							
History of hyperlipidemia	3.507	1.160–10.600	0.026							
History of congestive heart failure	0.061	0.004–0.827	0.036							

SBP, systolic blood pressure; ACS, acute coronary syndrome.

## 4. Discussion

A rapid AD diagnosis is crucial for medical and surgical therapy outcomes [20]. However, there is no single definitive diagnostic AD test that can be performed in the field that is non-invasive, rapid, easily accessible, and has high sensitivity and specificity. The final diagnosis of AD also depends on imaging techniques. Contrast-enhanced computed tomography angiography (CTA) is the most frequently used definitive diagnostic test for AD. The diagnostic time duration differs depending on the patient population, study site, etc. In previous studies, the time from the onset of symptoms to diagnosis ranged from 40.5 min to 84.4 h, and the time from hospital presentation to diagnosis ranged from 0.5 h to 25 h [2, 5, 6, 9, 10, 11, 19, 20, 21, 22, 23, 24, 25, 26, 27, 28, 29, 30, 31, 32, 33, 34, 35, 36, 37, 38, 39]. Many studies on AD diagnostic times have been based on the IRAD. The IRAD, established in 1996, is the largest contemporary study of AD and has collected data for patients with AD consecutively admitted to 56 tertiary care centers in 14 countries up to 2022 [6].

### 4.1 Demographic and Medical History Predictors

Several studies have enumerated the factors associated with a delayed diagnosis of AD [2, 20, 21, 31, 39, 40]. Demographic and medical history predictors included the female sex, age <70 yrs, North American versus European geographic location, being the initial AD presentation, history of congestive heart failure, history of hyperlipidemia, distressed communities index >60, walk-in visit to the ED, transfer from a non-tertiary care facility, and preoperative CA.

More women than men waited for more than 24 h before diagnosis, and this was attributed to atypical presentation symptoms. Women appear likely to experience less typical or less severe pain perception, with less frequent abrupt onset and more frequently observed alterations in consciousness, partly accounting for the longer delay in diagnosis [40]. Harris *et al*. [2] also reported that women were diagnosed more slowly.

Patients <70 years of age had a higher risk of delayed diagnosis. This association may be attributed to a lower index of diagnostic suspicion of spontaneous AD in patients with fewer strong age-related risk factors and comorbidities [20]. Furthermore, in agreement with previous studies, Lim *et al*. [39] found that patient age was associated with delayed diagnosis.

Raghupathy *et al*. [21] observed a significant delay in presentation and diagnosis of AD in a North American patient cohort compared to that in European cohorts. North Americans have a higher percentage of atypical presenting symptoms and signs, ECG findings that suggest acute ischemia, and a tendency toward more normal-appearing chest radiographs. This may have contributed to the delayed diagnosis. In addition, differences were observed in the choice of the initial imaging test for patients between North American and European IRAD centers. More European centers use computed tomography (CT) scans as the first diagnostic test, obtaining the most readily available imaging data to confirm the diagnosis accurately and rapidly.

Isselbacher *et al*. [31] reported that compared to initial AD, the time from the onset of symptoms to diagnosis was significantly shorter in patients with recurrent AD. Therefore, recurrent AD is a protective factor against delayed diagnoses, probably because patients with recurrent AD have a better understanding of the disease and can reach the hospital more quickly when recurrence occurs. Their history of AD may also help doctors make a rapid diagnosis.

A history of congestive heart failure (CHF) was observed to be associated with a decreased risk of delayed diagnoses for AD [31]. Conversely, the presentation of CHF significantly prolonged AD diagnosis [2]. A larger sample size is required for further validation of this finding. In contrast, a history of hyperlipidemia and residence in a high-distressed communities index (DCI) zip code were associated with an increased risk of diagnostic delay [39]. Hyperlipidemia may increase the clinical suspicion of ACS. The DCI is an aggregate measure of community-based socioeconomic status (SES). To reduce diagnostic delay, improving our understanding of the patient, the patient environment, and the healthcare system treating this condition will be critical.

### 4.2 Walk-in Visits to the ED

Walk-in visits to the ED were associated with a delayed diagnosis of AD [9]. The clinical manifestations of AD are diverse. If a patient with AAD presents to the ED with symptoms mimicking those of other diseases, the correct diagnosis may be missed or delayed. The walk-in mode of admission was also the strongest predictor of misdiagnosis in a study by Kurabayashi *et al* [11]. Although clinicians tend to regard walk-in patients as less likely to be seriously ill, the significance of the findings related to ED walk-in and delayed diagnosis of AD need to be remembered.

Strauss *et al*. [32] showed that patients with delayed diagnoses were more likely to be transferred from a referral hospital. Harris *et al*. [2] also found that delays in AD diagnosis occurred in patients transferred from non-tertiary hospitals. Owing to the high risk and complexity of AD, most non-tertiary hospitals are not equipped to treat AD, and the referral rate is extremely high, ranging from 68.2% to 79.0% [10, 21, 40, 41, 42]. The medical practice experience of clinicians, particularly related to aortic emergencies, may be especially relevant. It is not feasible to perform CTA for all ED patients presenting with chest or back pain, especially in non-tertiary hospitals. In a study by Pare *et al*. [28], patients with ascending AD who underwent emergency physician-focused cardiac ultrasound (EP FOCUS) were diagnosed more quickly. In addition, FOCUS is a rapid, noninvasive test, and Pare *et al*. [28] recommended that evaluation of the aorta be performed in patients with symptoms suggestive of AD. Improved physician awareness and recognition of AD and prompt transport are both important. Inter-hospital transfer requires coordination between hospitals, and a systematic approach to the diagnosis and management of AD will need to be developed and used as a reference. This includes the creation of regional networks where defined protocols allow for the most expedient diagnosis and transfer of patients with AD to Aortic Centers of Excellence for definitive treatment.

Ramanath *et al*. [23] observed significantly increased time delays from symptom onset to diagnosis during preoperative CA before the surgical repair of TAAAD. Fortunately, preoperative CA was not associated with significant changes in in-hospital or long-term mortality rates.

### 4.3 Atypical AD Presentations (Without Typical Symptoms or Hemodynamic Instability)

Patients presenting without typical symptoms of AD or hemodynamic instability are more likely to experience diagnostic delays and be initially treated for more common etiologies. The median interval from symptom onset to diagnosis was 29 h in AAD with no pain and 10 h in patients with pain [5]. When patients do not have typical pain, clinicians may not initially consider AD, and this delays the diagnosis. A delayed diagnosis of painless AD is probably responsible for the higher mortality rate observed in patients without pain. This is consistent with the results reported by Tolenaar *et al*. [25]. Furthermore, previous reports have shown that 6.4%–15% of patients with AAD presented without severe or worst-ever pain [5, 24]. Clinicians should be aware of this rare condition.

Chest and back pain, especially when abrupt or radiating, occurred more frequently in the early diagnosis group. In contrast, syncope was more prevalent in patients with a delayed diagnosis [32]. In a study by Vagnarelli *et al*. [29, 30], patients with back pain were identified earlier. Similarly, typical presenting symptoms, such as chest and back pain expedited the diagnostic process [39]. Diagnostic delays occurred in patients with atypical symptoms that were not abrupt or did not include chest, back, or any other pain [2]. Moreover, fever at presentation is not a common symptom of AD, and so may lead to an alternative diagnosis [2].

In a study by Rapezzi *et al*. [20], two strong clinical confounders appeared to be pleural effusion and dyspnea which were associated with a three to fourfold elevated risk of delayed diagnosis. In a study by Vagnarelli *et al*. [29, 30], the increased risk of diagnostic delay was also related to dyspnea and pleural effusion. These two clinical presentations may prompt clinicians to formulate a primary diagnostic hypothesis for pulmonary or cardiac diseases.

A low SBP (<105 mmHg) was associated with a significantly decreased risk of diagnostic delay [20]. Likewise, an admission SBP ≥105 mmHg delay diagnosis [2]. In another study, SBP <90 mmHg was associated with earlier recognition of AD [30]. Additionally, the presence of malperfusion syndrome expedited the diagnostic process [39]. Pulse deficits were protective from delayed diagnoses [2, 29]. In patients with life-threatening limb ischemia, shock, syncope, or altered consciousness, the diagnosis of AD was achieved more quickly because multiple diagnostic tests were conducted concurrently [2].

### 4.4 Abnormalities of Laboratory Testing

Troponin positivity and ACS-like ECG lead to delays in the diagnosis of AD [20]. This observation is consistent with the findings of reports by Vagnarelli *et al*. [30] and Du *et al*. [27]. An initial suspicion of ACS was the most common reason for a missed or delayed diagnosis of AD [43]. An ECG is routinely performed when patients present with chest pain. The incidence of AAD is far lower than that of ACS [3]. In the absence of a specific biomarker for AD, troponin positivity is used, given the high frequency of ACS among emergency patients and shared causal risk factors for AAD and ACS. Notably, in many cases of AD, electrocardiographic repolarization abnormalities and/or increased troponin levels reflect the coexistence of myocardial ischemia [20]. These findings emphasize the need for clinicians to suspect AD whenever plausible, even in cases where the initial diagnostic hypothesis is ACS. The American College of Cardiology/American Heart Association aortic guidelines suggest that clinicians should focus on high-risk conditions that place patients at risk as well as typical historical and examination features to diagnose AD earlier [43].

## 5. Conclusions

In conclusion, the time to AD diagnosis varies depending on the study site. Multiple factors result in significant delays in the diagnosis of AD. Educational efforts to improve physician awareness of both typical and atypical presentations of AD and prompt transport of patients with AD may reduce crucial time variables, particularly in non-tertiary hospitals with low exposure to aortic emergencies. It is also important to increase awareness of the disease among medical staff and patients. Limitations of this scoping review include the differences in the study design and patient characteristics between the articles. Moreover, as all included studies reported statistically significant results, no negative results were reported, which may indicate potential publication bias.

## Supplementary Material

Supplementary material associated with this article can be found, in the online version, at https://doi.org/10.31083/RCM33487.
